# MVA-Spike encoding the A subunit of dmLT safely enhances systemic and mucosal immune responses

**DOI:** 10.3389/fimmu.2026.1771410

**Published:** 2026-02-05

**Authors:** Stephan Rambichler, Ronny Kassub, Rodrigo Carrasco-León, Kerstin Lämmermann, Markus Feigl, Barbara Bathke, Clémentine Durand, Živa Fras, Alexander Heiseke, André Riedl, Andrea Koppius, Florian Brod, Mark Suter, Jürgen Hausmann, José Medina-Echeverz, Paul Chaplin, Hubertus Hochrein, Maria Hinterberger

**Affiliations:** 1 Bavarian Nordic GmbH, Planegg, Germany; 2 GlaxoSmithKline GmbH, München, Germany; 3 CatalYm GmbH, Planegg, Germany

**Keywords:** dmLT, double mutant heat labile toxin, modified vaccinia virus Ankara (MVA), mucosal adjuvant, vaccine

## Abstract

**Introduction:**

Mucosal immunity provides frontline protection at respiratory, gastrointestinal, and urogenital surfaces, where secretory IgA and tissue-resident T cells such as TH17 limit colonization and early replication of pathogens. Conventional parenteral vaccines typically induce robust systemic immunity but fail to elicit strong mucosal responses. Therefore, the development of safe and effective strategies to enhance mucosal immunity remains a key priority in vaccine research.

**Methods:**

We designed a modified vaccinia virus Ankara (MVA) construct expressing the double mutated heat-labile enterotoxin (dmLT) or only its double mutated A subunit (dmLT-A) together with Spike protein of SARS-CoV-2. C57BL/6 mice were immunized either intramuscularly or intranasally and immune responses as well as safety were monitored.

**Results:**

Here we show that encoding the A subunit of dmLT in MVA-Spike (MVA-Spike-dmLT-A) enhances systemic and mucosal immune responses after intramuscular or intranasal immunization compared to non-adjuvanted MVA-Spike. MVA-Spike-dmLT-A elicited a multifunctional T helper response including the induction of TH17 cells in spleen and lung. This was accompanied by the efficient generation of Spike-specific antibodies in blood and lung including IgA. Histological analysis revealed the formation of organized lung-associated lymphoid structures in mice immunized with MVA-Spike-dmLT-A. Importantly, MVA encoding the holotoxin dmLT led to a massive influx of immune cells and secretion of proinflammatory mediators in the lung resulting in significant weight loss after intranasal immunization. By contrast, MVA-Spike-dmLT-A was well tolerated and did not show any signs of toxicity.

**Conclusion:**

Our findings demonstrate that the A subunit of dmLT is a potent in-built adjuvant when expressed by MVA. It induces systemic and mucosal immune enhancement comparable to the full toxin mutant without any toxicity. Combining the strong immunogenic profile of MVA with the mucosal immune–modulating properties of the A subunit of dmLT represents a highly effective new vaccine platform.

## Introduction

Mucosal immunity plays a central role for defending the body against a wide range of pathogens that enter through mucosal surfaces, such as the respiratory, gastrointestinal, and urogenital tracts. These sites serve as major portals for infection, making the mucosal immune system the first line of defense. Secretory IgA antibodies, tissue-resident memory T cells including TH17 cells, and local innate immune mechanisms collaborate to limit early replication upon entry and to prevent pathogen colonization.

Traditional vaccines are often administered via parenteral routes, such as intramuscular or subcutaneous injection. While these routes commonly elicit robust systemic antibody and T cell responses, they frequently fall short in stimulating strong and durable immune protection at mucosal sites (1–3). Protective immunity against respiratory pathogens such as SARS-CoV-2 can benefit from strong mucosal responses - including secretory IgA and tissue-resident TH17/TH1 cells - in order to neutralize the virus at the entry site and reduce transmission (4, 5). This highlights the need for vaccine strategies that induce mucosal immunity and in addition provide systemic protection.

Administering vaccines via mucosal routes, such as oral or intranasal delivery, is an effective strategy to direct immune responses toward mucosal tissues and promote mucosa-specific immunity. Another approach to boost mucosal-type immune responses is the inclusion of specialized adjuvants that skew immunity toward TH17 cells and IgA production. Among the most promising are bacterial proteins like flagellin and the ADP-ribosylating enterotoxin-based adjuvants, such as cholera toxin or the heat-labile enterotoxin (LT) of *Escherichia coli* and its detoxified mutants (6, 7). LT is an AB_5_ toxin composed of an enzymatically active A subunit and a pentameric B subunit responsible for cell binding and entry via the ganglioside GM1 (8–12). In intestinal epithelial cells, LT binds GM1 receptors and undergoes retrograde transport to the endoplasmic reticulum, where the A subunit is cleaved into the enzymatically active A1 subunit. The A1 subunit is transported to the cytoplasm and activates adenylate cyclase, which causes an increase of intracellular cyclic AMP (cAMP) and ultimately leads to intestinal fluid secretion and diarrhea (7, 13–15). To reduce LT mediated adverse events, a detoxified double-mutant of LT (dmLT, LT R192G/L211A) was created. The two point mutations in the A subunit reduce ADP-ribosyltransferase activity, thereby lowering cAMP-mediated adverse effects while preserving its adjuvant properties (16, 17). LT and its mutants have been shown to act as powerful adjuvants that promote a multifaceted immune responses, including inflammatory TH1, TH2, and TH17 responses, cytotoxic T lymphocytes, and enhanced systemic and mucosal antibody production (18–21). Mechanistically, LT induces the maturation of antigen-presenting cells, including dendritic cells (DCs) and macrophages, via activation of cAMP-dependent Protein Kinase A (PKA) and Exchange Protein Directly Activated by cAMP (EPAC) signaling pathways (22). This signaling cascade leads to increased expression of MHC class II and the costimulatory molecules CD80 and CD86, as well as the production of pro-inflammatory cytokines including IL-1β, IL-6, IL-23, and TNF-α, which collectively promote TH17 cell differentiation (19). IL-23 in particular is known to enhance IL-17 production by T cells (23). Importantly, LT’s potent adjuvanticity can also increase CD8^+^ T-cell responses (18), complementing the predominant CD4^+^ TH17 skewing.

Despite its efficacy as an adjuvant, a major concern with LT or its mutants has been toxicity, especially when administered via the intranasal route. Intranasal vaccines adjuvanted with LT have previously caused adverse effects such as Bell’s palsy in human recipients, leading to the withdrawal of a nasally delivered influenza vaccine that included LT as adjuvant (24). Even a detoxified LT with a single mutation (mLT) showed noticeable safety issues in the clinic (25). The double-mutant dmLT is considerably safer and has demonstrated favorable results in clinical trials (17, 26–29). Nevertheless, inclusion of the full toxin, or even detoxified mutants, carries an increased risk of residual unfavorable reactogenicity (27, 30).

One strategy to improve safety is to modify the toxin by removing its cell-binding domain. Previous studies demonstrated that the isolated A subunit can function as an adjuvant on its own, promoting significant IgG2a, IgA, and TH17 responses to co-delivered antigens, but it needs to be applied at much higher doses since cell entry is inefficient without the B subunit (20, 31, 32). Encouragingly, using only the A subunit could circumvent many safety issues: the LT-A protein by itself has no measurable enterotoxicity or neurotoxicity in animal models (20, 31).

We hypothesized that by expressing the adjuvant from a viral vector, the need for a B subunit may be eliminated, as the adjuvant would be produced directly inside infected host cells. In this way, virus-encoded dmLT-A (A subunit only) could amplify the immune response (especially TH17 and IgA) through localized intracellular action, while reducing systemic exposure and toxicity. For this purpose, we employed our well-established vaccine vector modified vaccinia virus Ankara (MVA-BN^®^; Bavarian Nordic). MVA is a highly attenuated, replication deficient vaccinia strain with excellent immunogenicity and safety profile. It is a strong inducer of type I interferon responses promoting robust and durable humoral as well as cellular immunity against vector-encoded heterologous antigens (33–35). A key safety attribute of MVA-BN^®^ is its inability to replicate in human cells, as productive replication is largely restricted to embryonic avian cells (36). Extensive preclinical and clinical data have established MVA as a safe and immunogenic vaccine platform, leading to the approval of the proprietary MVA-BN^®^ as a non-replicating vaccine against smallpox and monkeypox (37). The genetic stability of the MVA genome as well as its high capacity for incorporation of foreign DNA allows for the expression of multiple pathogen-derived antigens or immunostimulatory components in a single vector. Furthermore, MVA has been described to induce local T cell and B cell responses upon intranasal application (38).

In this study, MVA was engineered to co-express SARS-CoV-2 Spike with either dmLT or only its A subunit. Both constructs elicited substantially stronger and more diverse T cell responses, including robust TH17 differentiation and higher antibody titers than non-adjuvanted MVA after parenteral or intranasal immunization. Intranasal delivery further enhanced antigen-specific IgA and respiratory TH17 cells. Unlike MVA-dmLT, which caused noticeable injection-site inflammation and transient weight loss after intranasal administration, MVA-Spike-dmLT-A induced no detectable adverse effects. Importantly, the dmLT-A construct matched the immunogenicity of the full toxin dmLT. Thus, integrating the A subunit of dmLT into MVA enhances and broadens vaccine responses, offering strong T cell activation, TH17 polarization, and mucosal IgA without compromising safety.

## Results

### Immunogenicity and toxicity of MVA-OVA expressing dmLT holotoxin

**Figure 1:**
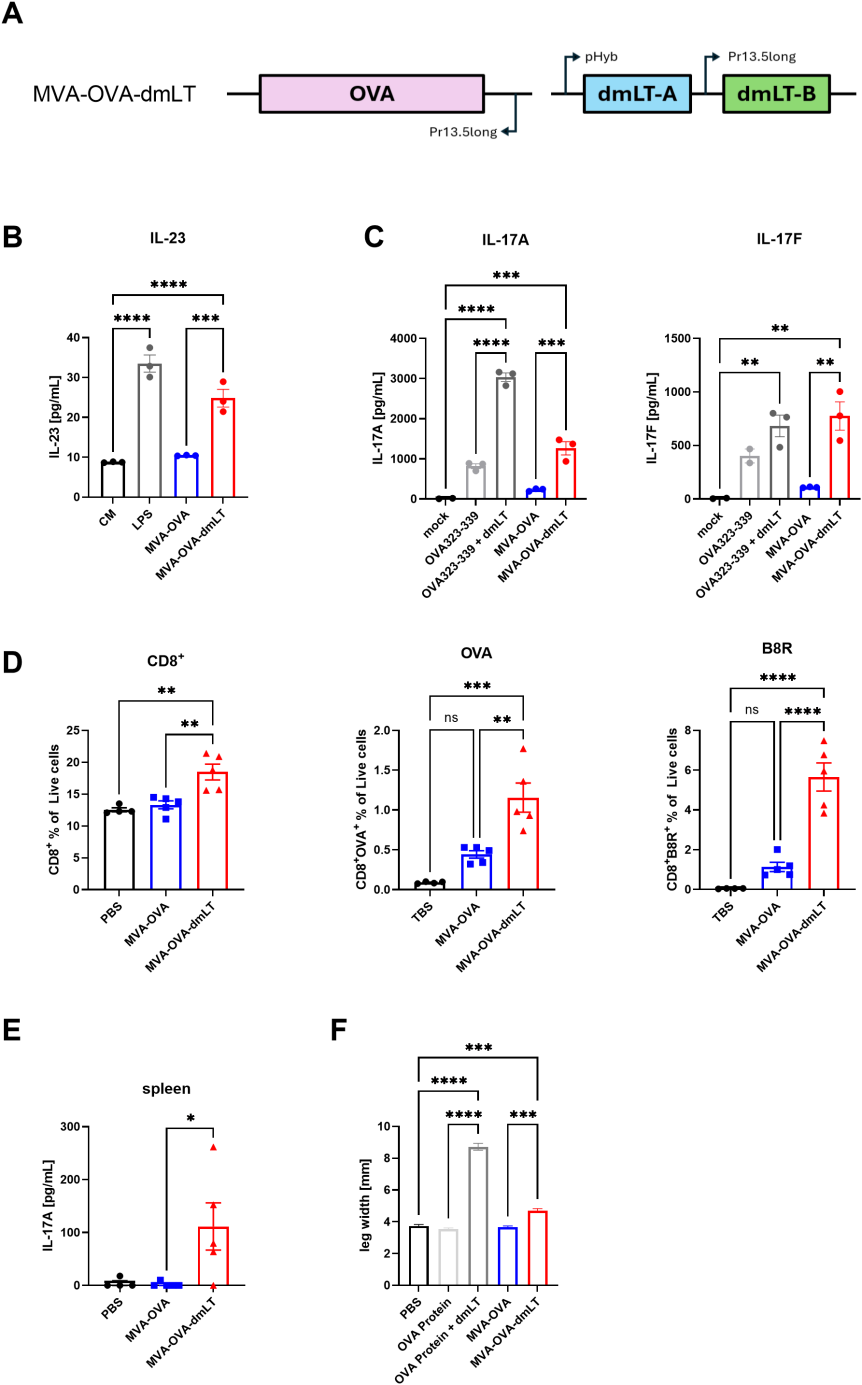
**(A)** Schematic representation of recombinant MVA encoding Ovalbumin (OVA) and the adjuvant dmLT. The expression of OVA and of the B subunit of dmLT are controlled by the p13.5-long promoter (39), and expression of dmLT-A is controlled by the early/late hybrid promoter pHyb (40). **(B)** GM-CSF BMDCs were incubated with 4 MOI MVA-OVA, 4 MOI MVA-OVA-dmLT, and 1 µg/mL LPS as a positive control for 48 hours before analysis of IL-23 in supernatants by ELISA. **(C)** GM-CSF BMDCs were incubated with 1 µg/mL OVA peptide, 1 µg/mL OVA peptide + 1 µg/mL dmLT, 2 MOI MVA-OVA, or 2 MOI MVA-OVA-dmLT for 2 hours, then co-cultured with OT-II CD4^+^ T cells for 4 days. Cytokine levels in the supernatants were determined by Luminex (IL-17F) or ELISA (IL-17A). **(D)** Single cells were isolated from the spleen 7 days after prime immunization of C57BL/6 mice and stained with B8R (TSYKFESV)-specific or OVA (SIINFEKL)-specific dextramers, along with fluorescently labeled antibodies, and analyzed by flow cytometry. Antigen-specific CD8 T cells were identified as Live^+^ Single CD4^neg^ CD8^+^ Dextramer^+^. **(E)** Splenocytes from mice (as in D) were restimulated with OVA protein for 72 h at 37°C. Supernatants were analyzed for cytokine concentrations via Luminex (IL-17A). **(F)** Thickness of the injected hind limb was measured 1 day after intramuscular immunization with 1×10^8^ TCID_50_ MVA-OVA, 1×10^8^ TCID_50_ MVA-OVA-dmLT, 10 µg OVA protein, or 10 µg OVA protein + 5 µg dmLT adjuvant. Data are shown as mean ± SEM. One-way ANOVA was performed. ns, non-significant; *p < 0,05; **p < 0,01; ***p < 0,001; ****p < 0,0001.

To enhance and broaden the immunogenic profile of MVA - both in magnitude and functional diversity - we engineered a recombinant MVA expressing the detoxified bacterial enterotoxin (dmLT). The dmLT subunits A and B were inserted into the MVA genome (insertion site IGR 148/149) under different poxviral promoters, and Ovalbumin (OVA) was included as a model antigen in a separate insertion site (IGR 44/45) (**Figure 1A**). A well-described feature of LT and its detoxified variants is the activation of myeloid cells, which leads to secretion of IL-23 and thereby triggers the induction of TH17 cells (19, 23). MVA-encoded dmLT was tested in bone marrow derived dendritic cells (BMDCs) for its ability to induce IL-23. MVA-OVA-dmLT but not MVA-OVA induced IL-23 production by GM-CSF BMDCs (**Figure 1B**). Furthermore, we tested whether MVA-OVA or MVA-OVA-dmLT-infected BMDCs could promote TH17 differentiation *in vitro*. For this, BMDCs were infected with MVA-OVA or MVA-OVA-dmLT; OVA peptide with or without dmLT adjuvant served as a positive control. OVA-specific naïve OT-II CD4^+^ T cells were added to the BMDCs and co-cultured for 4 days, followed by analysis of the supernatants for IL-17A and IL-17F levels. OVA peptide adjuvanted with dmLT elicited IL-17A and IL-17F production by T cells, whereas OVA peptide without adjuvant did not. Likewise, infection of BMDCs with MVA-OVA-dmLT but not MVA-OVA prior to T cell co-culture elicited robust IL-17A and IL-17F production by OT-II cells (**Figure 1C**).

Next, we evaluated the immunogenicity of the MVA constructs *in vivo* in C57BL/6 mice. Mice were injected intramuscularly with 5×10^7^ TCID_50_ of MVA-OVA or MVA-OVA-dmLT. We observed an increase in the frequency of CD8^+^ T cells in the spleens of mice injected with MVA-OVA-dmLT compared to MVA-OVA seven days after immunization (**Figure 1D**). Moreover, among these CD8^+^ T cells a higher percentage of OVA- and MVA (B8R)-specific cells was found in mice immunized with MVA-OVA-dmLT (**Figure 1D**). Complementary to dextramer staining, splenocytes were restimulated with OVA protein *ex vivo*. After 72 h of restimulation, the supernatants were collected and assessed by Luminex to quantify IL-17A secreted by OVA-specific T cells. IL-17A was detected in the supernatants of splenocytes isolated from MVA-OVA-dmLT-immunized mice, but not in those from control groups, showing that the MVA-OVA-dmLT effectively promoted TH17 responses (**Figure 1E**).

Since LT has a history of toxic complications, we closely monitored the mice after intramuscular immunization with MVA-OVA-dmLT. C57BL/6 mice were immunized intramuscularly with MVA expressing OVA with or without dmLT. Additionally, groups of mice were immunized with OVA protein ± dmLT adjuvant as controls, and mice injected with PBS served as baseline controls. We detected significant swelling of the injected hind limb in the OVA protein + dmLT adjuvant group 1 day after immunization (**Figure 1F**). We also observed mild swelling in MVA-OVA-dmLT-immunized mice, although to a significantly lower degree (**Figure 1F**). Thus, residual toxicity of dmLT led to local inflammation at the injection site, irrespective of whether the adjuvant was delivered as a protein or encoded in MVA.

### Design and characterization of MVA-Spike expressing the A subunit of dmLT

**Figure 2:**
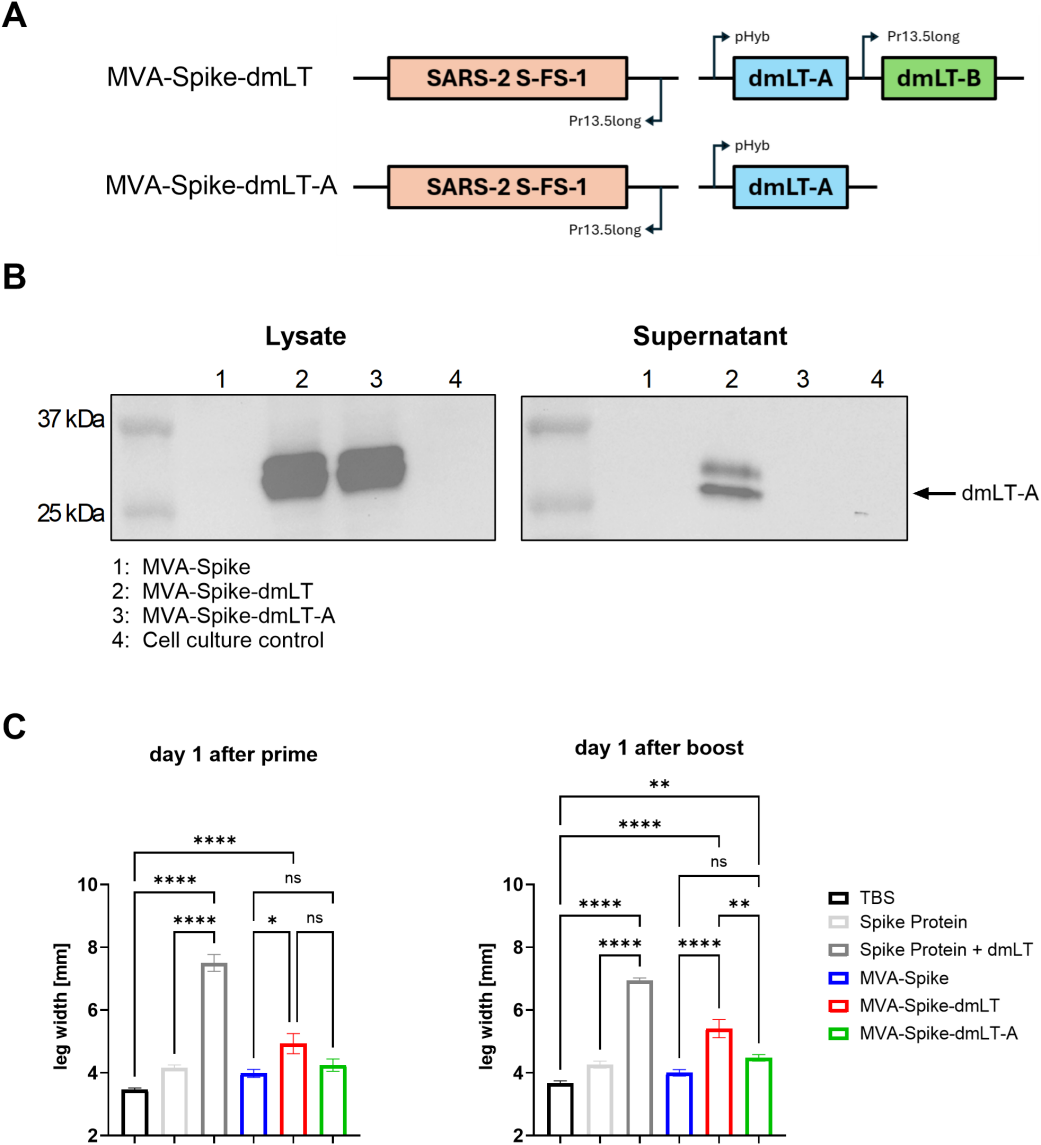
**(A)** Schematic representation of recombinant MVA encoding SARS-CoV-2 Spike and dmLT, or only the A subunit of dmLT. SARS-CoV-2 Spike and dmLT-B expression are controlled by the p13.5^long^ promoter, and expression of dmLT-A is controlled by the early/late hybrid promoter pHyb. **(B)** Vero cells were infected with 5 InfU per cell of MVA-Spike, MVA-Spike-dmLT, or MVA-Spike-dmLT-A for 1 hour. Cell lysates and cell culture supernatants were harvested 23 hours after infection and analyzed by Western blot for dmLT-A. **(C)** Wild-type C57BL/6 mice were primed intramuscularly on day 0 and boosted on day 21 with TBS (control), 5 µg Spike protein, 5 µg Spike protein + 1 µg dmLT adjuvant, 1×10^8^ InfU MVA-Spike, 1×10^8^ InfU MVA-Spike-dmLT or 1×10^8^ InfU MVA-Spike-dmLT-A. The thickness of both hind legs was measured 1 day after prime or boost immunization using a caliper. Data are shown as mean ± SEM. One-way ANOVA was performed. ns = non-significant; *p < 0,05; **p < 0,01; ****p < 0,0001.

Previous work showed that the A subunit of LT can be used as an adjuvant but needs to be applied at much higher doses since cell entry is inhibited in the absence of the B subunit (20, 31). Based on this, we hypothesized that the B subunit may be redundant for the adjuvant effect when the A subunit alone was expressed intracellularly by MVA. Moreover, removing the B subunit could decrease effects on bystander cells due to less efficient uptake of the A subunit into non-infected cells, which could eliminate the side effects observed with dmLT. In addition to removing the B subunit, we decided to use the Spike protein of SARS-CoV-2 as a more clinically relevant antigen instead of OVA (**Figure 2A**). Spike was inserted into the MVA genome in the insertion site IGR 64/65 under the control of the Pr13.5long promoter. DmLT subunit A and/or B were inserted in the insertion site IGR 88/89 under the control of the pHyb promoter and Pr13.5long promoter, respectively (**Figure 2A**). To verify the design of the constructs, we determined expression of the dmLT-A and dmLT-B subunits 24 h after infection of Vero cells with the recombinant MVAs. We detected both dmLT-A and dmLT-B subunits in cell lysates and supernatants after infection with MVA-Spike-dmLT (**Figure 2B** and **Supplementary Figure 1**). Interestingly, after infection of cells with MVA expressing only the dmLT-A subunit, dmLT-A was detected exclusively in the cell lysate but not in the supernatant, indicating that extracellular release only occurs when the complete toxin is assembled.

Next, C57BL/6 mice were prime-boost immunized intramuscularly with MVA-Spike-dmLT, MVA-Spike-dmLT-A, or MVA encoding Spike alone and potential leg swelling was assessed. Spike protein with or without dmLT adjuvant was included as positive control, and mice injected with TBS served as baseline controls. Similar to the results shown in **Figure 1F**, we detected significant swelling of the injected leg in the Spike protein + dmLT adjuvant group, even with a 5-fold lower dmLT adjuvant dose compared to the previous experiment (**Figure 2C**). We also observed mild swelling in MVA-Spike-dmLT immunized mice. In contrast, no significant swelling was induced in mice immunized with MVA-Spike-dmLT-A which was comparable to MVA-Spike alone (**Figure 2C**).

### MVA-Spike-dmLT-A elevates humoral and cellular immune responses including TH17

**Figure 3:**
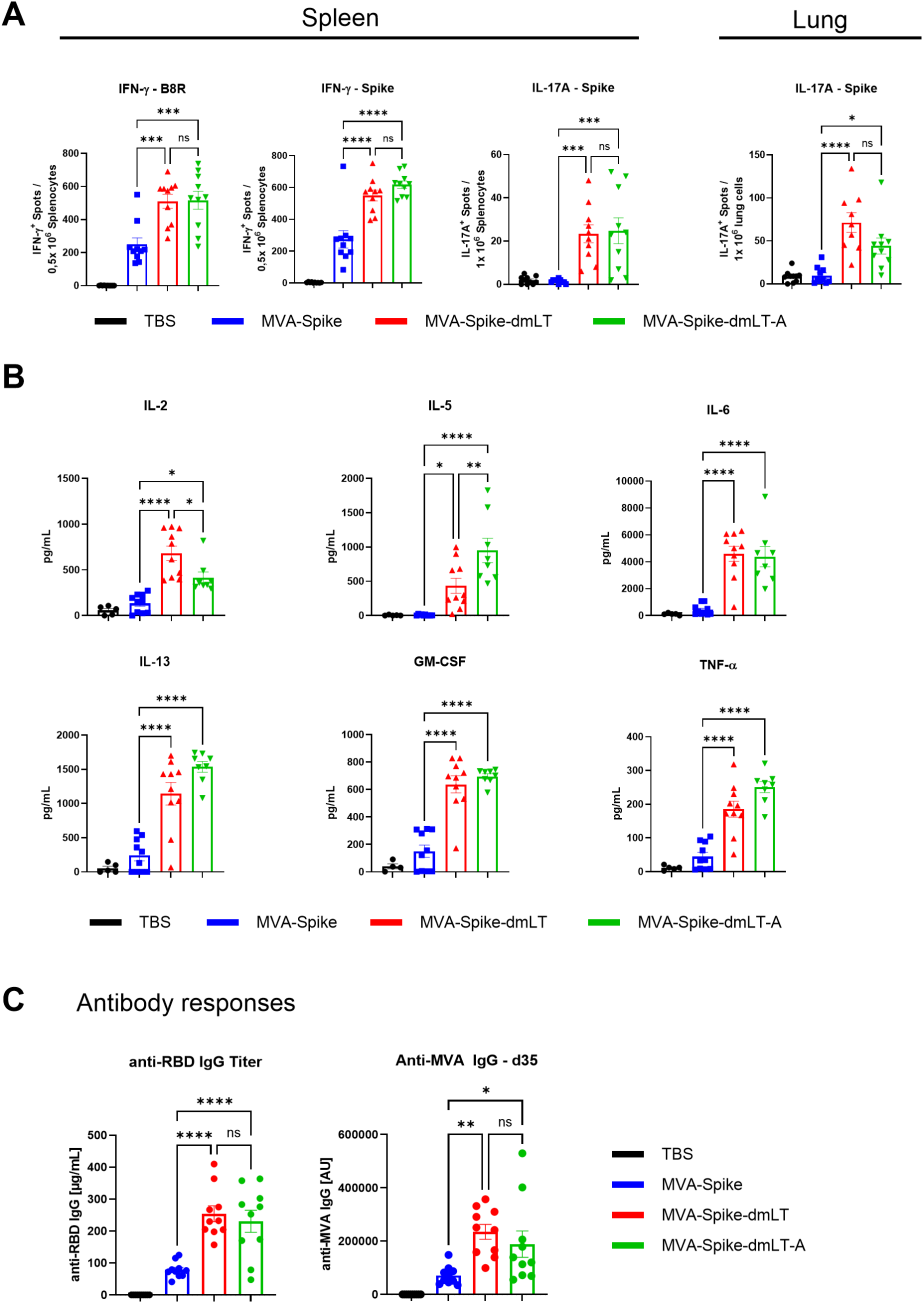
Wild-type C57BL/6J mice were immunized intramuscularly in one hind leg with 1×10^8^ InfU MVA-Spike, 1×10^8^ InfU MVA-Spike-dmLT, 1×10^8^ InfU MVA-Spike-dmLT-A, or TBS (control). **(A)** Fourteen days after prime immunization, splenocytes were isolated and restimulated with SARS-CoV-2 Spike peptide pool (Spike) or MVA B8R peptide (TSYKFESV) on IFN-γ ELISPOT plates. IFN-γ-producing cells were revealed with biotinylated anti-IFN-γ antibody, followed by streptavidin-HRP and AEC substrate. To detect IL-17A–producing cells, single cells from the spleen or lung were restimulated with Spike peptide pool on IL-17A ELISPOT plates; spots were developed with biotinylated anti-IL-17A antibody, followed by streptavidin-ALP and BCIP/NBT-plus substrate (Mabtech). Spots were counted using an ELISPOT reader. **(B)** Splenocytes (from day 14, as in A) were restimulated with recombinant SARS-CoV-2 Spike protein for 3 days at 37°C. Supernatants were analyzed for cytokine concentrations via Luminex (measuring representative TH1 (IL-2, TNF-α), TH2 (IL-5, IL-13), and TH17/TH-GM (GM-CSF) cytokines, as well as IL-6). **(C)** Sera were collected on day 35 (two weeks after a boost on day 21) and analyzed for anti-Spike RBD IgG and anti-MVA IgG by ELISA. RBD-specific IgG titers were quantified against a standard monoclonal anti-RBD antibody. MVA-specific IgG titers were determined by coating plates with inactivated MVA and performing serial serum dilutions. Titers are presented as endpoint dilution values (samples below the detection cutoff were assigned an arbitrary value of 1). Data are shown as mean ± SEM. One-way ANOVA was performed. ns, non-significant; *p < 0,05; **p < 0,01; ***p < 0,001; ****p < 0,0001.

Having observed that encoding only the A subunit (MVA-Spike-dmLT-A) prevented the leg swelling induced by full dmLT, we next investigated whether dmLT-A expressed by MVA could still deliver the full spectrum of immunogenic effects associated with dmLT. Mice were immunized intramuscularly with 1×10^8^ InfU of MVA-Spike, MVA-Spike-dmLT, or MVA-Spike-dmLT-A, and TBS as control group. Antigen-specific T cells were analyzed in spleen 14 days after immunization by ELISPOT assay. Both MVA-Spike-dmLT and MVA-Spike-dmLT-A elicited higher Spike-specific and MVA-specific (B8R) IFN-γ responses compared to MVA-Spike (**Figure 3A**). Additionally, we also detected SARS-CoV-2 Spike-specific IL-17A-producing cells in mice immunized with MVA-Spike-dmLT and MVA-Spike-dmLT-A (**Figure 3A**), demonstrating successful induction of TH17 responses *in vivo*. Notably, robust induction of TH17 cells by MVA-Spike-dmLT or MVA-Spike-dmLT-A was also observed in the lung, demonstrating that intramuscular immunization elicits TH17 responses at distant mucosal entry sites (**Figure 3A**). To test whether MVA adjuvanted with dmLT or dmLT-A would induce a diverse CD4^+^ T helper cell profile, we restimulated splenocytes with SARS-CoV-2 Spike protein for 72 hours 14 days after immunization and measured a panel of cytokines. The supernatants were analyzed by Luminex for TH1 (IL-2, TNF-α), TH2 (IL-5, IL-13), and TH17/TH-GM (GM-CSF) cytokines (**Figure 3B**) (41, 42). Mice immunized with MVA-Spike-dmLT or MVA-Spike-dmLT-A produced significantly higher levels of TH1-, TH2-, and TH17-associated cytokines as well as IL-6 compared to mice immunized with MVA-Spike alone (**Figure 3B**). Finally, we evaluated SARS-CoV-2 Spike RBD-specific and MVA-specific antibody responses in the serum two weeks after boost immunization. We observed approximately 3-fold higher RBD-specific and MVA-specific IgG titers in mice immunized with MVA-Spike-dmLT and MVA-Spike-dmLT-A compared to MVA-Spike (**Figure 3C**).

Together, removal of the B subunit in MVA-Spike-dmLT-A led to the development of a safer vaccine platform that maintains adjuvant activity, enhances T and B cell responses - including TH17 immunity in the lung - and broadens CD4^+^ cytokine profiles without inducing injection-site inflammation (**Figure 2**).

### Mucosal immunity against SARS-CoV-2 enhanced by MVA-Spike-dmLT-A

**Figure 4:**
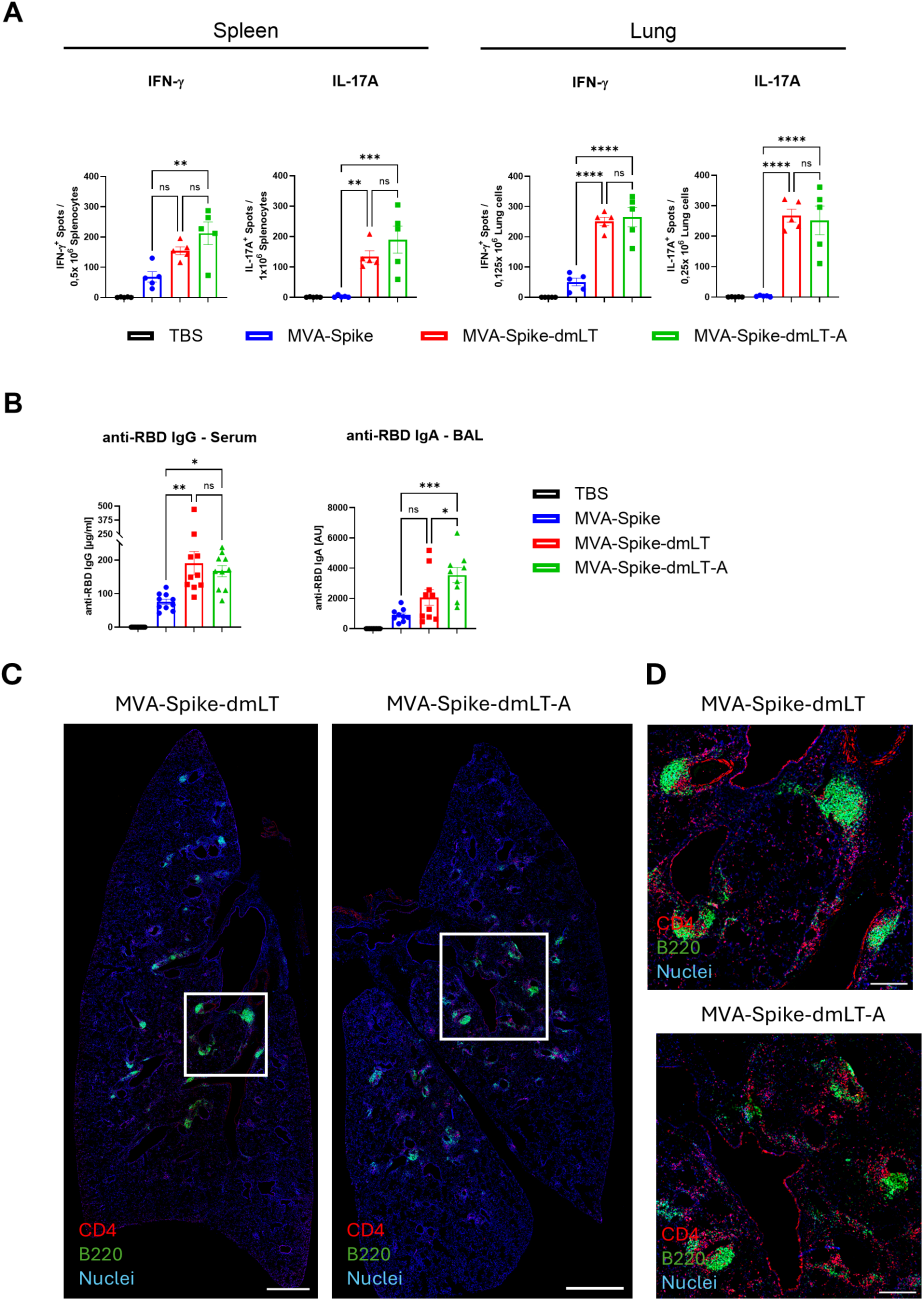
Wild-type C57BL/6J mice were primed intranasally with 1×10^7^ InfU MVA-Spike, 1×10^7^ InfU MVA-Spike-dmLT, 1×10^7^ InfU MVA-Spike-dmLT-A, or TBS (control). **(A)** Single-cell suspensions from lung and spleen were isolated 14 days after immunization and restimulated with SARS-CoV-2 Spike peptide pool on IFN-γ ELISPOT plates. IFN-γ-producing spots were counted. For IL-17A ELISPOT, cells were restimulated with Spike peptide pool and IL-17A-producing spots were enumerated. Data are presented as spot-forming cells (SFC) per 10^6^ cells. **(B)** Mice were boosted on day 21 via the same intranasal route. Sera and BAL were collected on day 35. Sera were analyzed for anti-RBD IgG by ELISA. BAL fluid was analyzed for anti-RBD IgA by ELISA. Antibody titers are expressed as arbitrary units (AU) using a reference standard, calculated with a 4 parameter logistic fit curve at OD 0,3 (samples below the cutoff were assigned an AU of 1). **(C, D)** Mice were primed intranasally with 2,5 × 10^7^ InfU MVA-Spike, 2,5 × 10^7^ InfU MVA-Spike-dmLT, 2,5 × 10^7^ InfU MVA-Spike-dmLT-A, or TBS (control) (n = 3 per group). Lungs were isolated 12 days after prime immunization for analysis by immunofluorescence microscopy. **(C)** Representative 20× tile scan images of immunohistochemically stained lung sections show CD4 (red), B220 (green), and nuclei (blue). Scale bar = 1000 µm **(D)** Close-up views of selected areas from the tile scan. Scale bar = 250 µm. Data are shown as mean ± SEM. One-way ANOVA was performed. ns, non-significant; *p < 0,05; **p < 0,01; ***p < 0,001; ****p < 0,0001.

Since dmLT has been identified as a potent mucosal adjuvant that promotes TH17 cell induction and enhances antigen-specific IgA responses (20, 43), we next evaluated the immunogenicity of mucosally delivered MVA (i.n.) expressing dmLT-A. We immunized C57BL/6 mice intranasally with 1×10^7^ InfU of MVA-Spike, MVA-Spike-dmLT, or MVA-Spike-dmLT-A as well as TBS as a control. A relatively low dose was chosen to avoid adverse effects, since we observed swelling at the injection site with a dose of 1×10^8^ InfU in the intramuscular setting (**Figure 2C**). Fourteen days after immunization, antigen-specific T cells in the lung and spleen were analyzed by ELISPOT. Both MVA-Spike-dmLT and MVA-Spike-dmLT-A induced higher numbers of IFN-γ spot-forming cells in single-cell suspensions from the lung and spleen after restimulation with either the B8R peptide or a SARS-CoV-2 Spike peptide pool, compared to MVA-Spike (**Figure 4A**, **Supplementary Figure 2A**). Consistent with our intramuscular results, we detected a striking increase in Spike-specific IL-17A-producing cells in both the lung and spleen of mice immunized intranasally with MVA-Spike-dmLT or MVA-Spike-dmLT-A (**Figure 4A**). Next, we analyzed Spike RBD-specific antibody responses in the serum by ELISA two weeks after boost immunization. RBD-specific IgG levels increased in mice immunized intranasally with MVA-Spike-dmLT and MVA-Spike-dmLT-A compared to MVA-Spike (**Figure 4B**). Finally, we measured mucosal antibody responses by determining RBD-specific IgA levels in BAL fluid two weeks after the boost immunization. We detected a significant boost of anti-RBD IgA titers in the BAL of mice immunized with MVA-Spike-dmLT-A compared to mice immunized with MVA-Spike (**Figure 4B**).

To better characterize local immune responses elicited by our vaccine platform, we performed immunohistochemical analysis of perfused lungs 12 days after intranasal immunization. Previous studies have demonstrated that inducible Bronchus-Associated Lymphoid Tissue (iBALT) can develop in the lung in response to certain viral infections or vaccinations and that TH17 cell are involved in the formation of iBALT (44–47). Accordingly, we performed immunostaining of lung sections to detect B cells (B220) and CD4^+^ T cells (CD4) 12 days after immunization. Whereas lungs immunized with MVA-Spike displayed only sporadic T and B cell clusters (**Supplementary Figure 2B**), intranasal immunization with MVA-Spike-dmLT or MVA-Spike-dmLT-A induced consistent iBALT formation across all mice analyzed based on multiple lung sections per animal (**Figures 4C, D**).

### Excellent safety profile of intranasal MVA-Spike-dmLT-A vaccination

**Figure 5:**
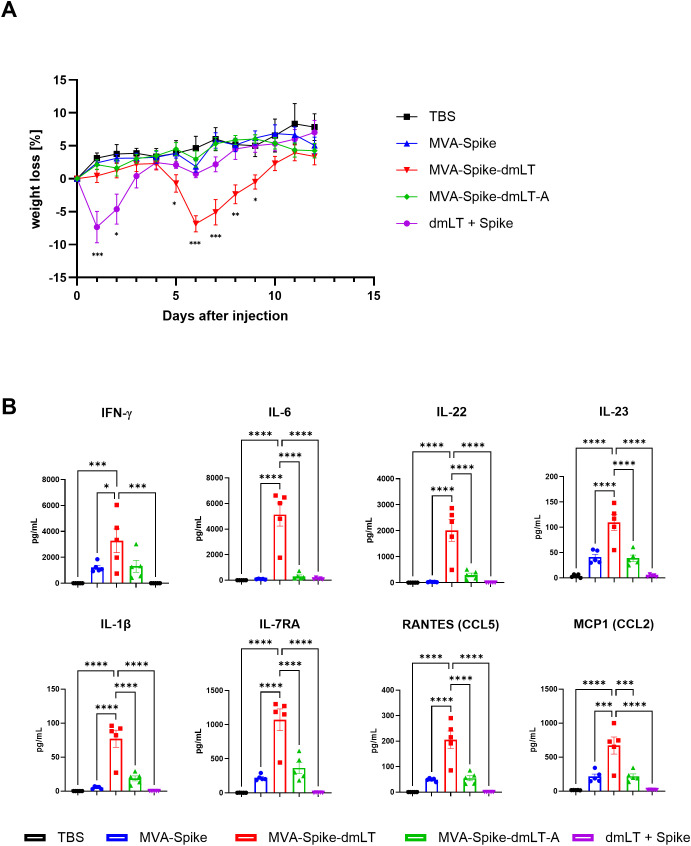
Wild-type C57BL/6J mice were primed intranasally with 2,5×10^7^ InfU MVA-Spike, 2,5×10^7^ InfU MVA-Spike-dmLT, 2,5×10^7^ InfU MVA-Spike-dmLT-A, 5 µg dmLT protein + 5 µg SARS-CoV-2 Spike protein, or TBS as control. **(A)** Mice were weighed daily; weight change is plotted as percentage of starting weight over 12 days post-immunization. **(B)** BAL was collected 5 days after immunization and analyzed by Luminex using a 48-plex cytokine/chemokine panel. Selected pro-inflammatory and TH17-related factors are shown (pg/mL). Data are shown as mean ± SEM (n = 4–5 per group for cytokine analysis). One-way ANOVA was performed. *p < 0,05; **p < 0,01; ***p < 0,001; ****p < 0,0001.

Given the absence of adverse effects following intranasal immunization of mice, we next investigated whether higher doses of dmLT- or dmLT-A-adjuvanted MVA would also be well tolerated via the intranasal route. We immunized mice intranasally with 2,5×10^7^ InfU of MVA-Spike, MVA-Spike-dmLT, or MVA-Spike-dmLT-A. Additionally, we included a group that received 5 µg dmLT protein mixed with 5 µg recombinant SARS-CoV-2 Spike protein to mimic a conventional protein subunit vaccine with adjuvant. TBS immunization served as the baseline control. Mice were monitored daily, scored for clinical appearance and behavior, and weighed to detect any weight changes following immunization. Mice in the TBS and MVA-Spike groups steadily gained weight over the two-week observation period (**Figure 5A**). In contrast, mice that received intranasal Spike protein + dmLT showed a transient weight loss starting shortly after immunization but recovered to normal weight by day 4 (**Figure 5A**). MVA-Spike-dmLT-immunized mice initially appeared normal but began to lose weight around day 5 post-immunization (**Figure 5A**). The weight loss in this group peaked around day 6, after which the mice steadily regained weight, approaching the weight of control groups by the end of the experiment on day 12 (**Figure 5A**). Strikingly, mice that received MVA-Spike-dmLT-A *i.n.* did not show any signs adverse reactions to the vaccine and gained weight comparable to the TBS immunized group. Apart from the observed weight changes, no other overt clinical symptoms were noted during the monitoring period for any group.

### Differences in lung immune cell infiltration and chemokine profile between MVA expressing dmLT or dmLT-A

To gain insight into the underlying cause of the transient weight loss observed in MVA-Spike-dmLT-immunized mice, we collected BAL fluid on day 5 after the prime immunization -corresponding to the onset of weight loss in this group - and analyzed cytokine and chemokine levels using a 48-plex Luminex assay. We detected an increase in many inflammatory analytes in groups that received *i.n.* MVA immunization. However, the profile of MVA-Spike-dmLT immunized mice was markedly distinct, exhibiting by far the highest levels of pro-inflammatory cytokines (**Figure 5B** and **Supplementary Figure 3**). Among the elevated factors were pro-inflammatory cytokines such as IL-6, IL-1β, and IFN-γ, as well as chemokines involved in recruitment of inflammatory cells (*e.g.*, RANTES/CCL5 and MCP-1/CCL2) (**Figure 5B**). Notably, several cytokines associated with TH17 responses, including IL-1β, IL-22, IL-23, and IL-17A, were uniquely or disproportionately elevated in the MVA-Spike-dmLT group at this early time point. In contrast, these TH17-related cytokines were only slightly elevated in mice immunized with MVA-Spike-dmLT-A, even though by day 14 both MVA-Spike-dmLT and MVA-Spike-dmLT-A induced similar levels of TH17 cells (**Figure 4A**).

**Figure 6:**
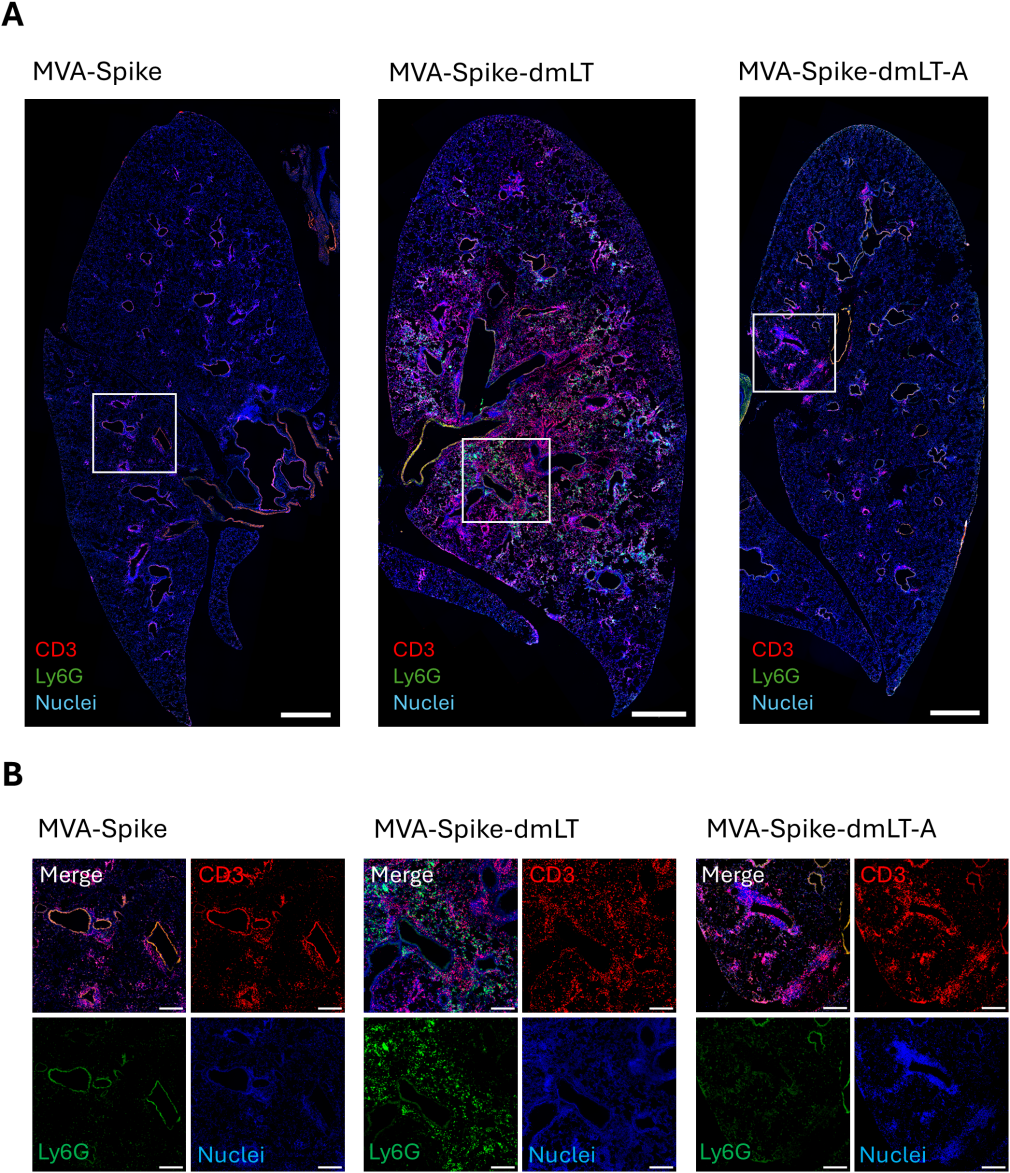
Mice were primed intranasally with 2,5 × 10^7^ InfU MVA-Spike, 2,5 × 10^7^ InfU MVA-Spike-dmLT, 2,5 × 10^7^ InfU MVA-Spike-dmLT-A, or TBS (control. See **Supplementary Figure 4**) (n = 3 per group). Lungs were isolated 5 days after immunization for immunofluorescence microscopy. **(A)** Representative 20x tile scan images of immunohistochemically stained lung sections show CD3 (red), Ly6G (green), and nuclei (blue). Scale bar = 1000 µm **(B)** Close-up views of representative lung sections highlighting immune cell infiltration. Merged images are shown alongside single-channel signals for CD3 (red), Ly6G (green), and nuclei (blue). Scale bar = 250 µm.

In view of these extensive changes in the proinflammatory cytokine and chemokine profile of MVA-dmLT immunized mice, we performed immunohistochemical analysis to detect potential cell infiltration into the lung tissue. Mice were perfused 5 days after *i.n.* immunization with MVA-Spike, MVA-Spike-dmLT and MVA-Spike-dmLT-A and tissue sections were stained for neutrophils (Ly6G) and T cells (CD3). MVA-Spike and MVA-Spike-dmLT-A hardly induced any infiltration of T cells or neutrophils. In contrast, a massive infiltration of neutrophils and T cells was visible throughout the whole lung tissues in MVA-Spike-dmLT immunized mice, possibly explaining the high proinflammatory response in the lung accompanied by the observed weight loss (**Figures 6A, B** and **Supplementary Figure 4**). These data indicate that MVA-Spike-dmLT-A was well tolerated after intranasal immunization, while MVA-Spike-dmLT caused transient weight loss and lung inflammation. Thus, removing the B subunit markedly improved the safety of the adjuvanted MVA-Spike-dmLT-A vaccine for mucosal delivery.

## Discussion

Our study demonstrates that an MVA-vectored vaccine can be effectively enhanced by incorporating an encoded mucosal adjuvant, resulting in substantially stronger and broader immune responses. Both the detoxified complete LT (dmLT) and the A subunit alone (dmLT-A) significantly boosted TH17 polarization in the spleen and lung, CD8^+^ T cell expansion against the encoded antigen and the vaccine platform, and antigen-specific antibody production, including mucosal IgA after intranasal immunization. Importantly, expression of the dmLT-A subunit alone retained full immunostimulatory activity while eliminating the adverse effects linked to dmLT.

We found that MVA-encoded dmLT potently activates DCs, as evidenced by IL-23 secretion, and drives naïve CD4^+^ T cells toward a TH17 phenotype *in vitro* (**Figures 1B, C**). This is in line with prior studies showing that LT and its mutants activate DCs to produce IL-1β and IL-23, which are key cytokines for TH17 differentiation (19, 23). Consequently, MVA-OVA-dmLT induced TH17 cells and increased antigen-specific IL-17A production both *in vitro* and *in vivo*. In addition to CD4^+^ T cell skewing, we observed that MVA-dmLT enhanced the magnitude of the CD8^+^ T cell response specific to both the model antigen (OVA) and the viral vector itself (B8R). This broad effect is consistent with the known ability of LT adjuvants to promote robust cytotoxic T lymphocyte responses (18). These findings validate the concept that an *in situ* expressed adjuvant can significantly improve an MVA vaccine’s performance.

A key question was whether the A subunit alone, without the B subunit, could confer similar immune benefits. Previous work indicated that the A subunit of LT can directly act on antigen-presenting cells and promote TH17 and IgA responses, but only when provided at relatively high concentrations (20, 31, 32). The requirement for a high LT-A dose when delivered exogenously is likely a result of the inefficient uptake of the LT-A subunit as well as the rapid proteolytic degradation of the A subunit in the absence of the B subunit (20). By delivering dmLT-A via the MVA vector, we effectively bypassed the need for extracellular toxin uptake: the adjuvant is made inside infected cells, which allows it to trigger innate immune pathways, such as inflammasome activation and cytokine release, from within. Strikingly, MVA-Spike-dmLT-A elicited immune responses nearly indistinguishable from those induced by MVA-Spike-dmLT.

Both adjuvanting strategies increased Spike-specific IFN-γ^+^ T cells (indicative of enhanced TH1 and CD8 responses), induced TH17 responses, and boosted serum IgG levels (**Figures 3A, C**). Furthermore, both MVA-Spike-dmLT and MVA-Spike-dmLT-A broadened the CD4^+^ T cell response profile, evidenced by higher production of not only TH17 cytokines but also TH1 (IL-2, TNF-α) and TH2 cytokines (IL-5, IL-13) upon antigen restimulation (**Figure 3B**). This shows that the adjuvant effects of MVA-encoded dmLT-A extend beyond just TH17 skewing, promoting a polyfunctional helper T cell response (19), which could be beneficial for overall protective immunity. Interestingly, not only intranasal immunization with MVA-Spike-dmLT-A but also intramuscular vaccination induced TH17 responses in the lung (**Figure 3A**). The induction of TH17 cells at the site of mucosal pathogen entry, even after parenteral immunization, may offer a significant advantage by strengthening local immune defenses and improving protection against mucosal pathogens (48, 49). We also showed that intranasal delivery of MVA-Spike-dmLT and MVA-Spike-dmLT-A led to the formation of induced bronchus-associated lymphoid tissues (iBALT) as indicated by the numerous organized B and CD4 T cell clusters in lungs 12 days after *i.n.* immunization. iBALTs serve as the mucosal counterparts of secondary lymphoid tissues and, as such, represent optimal sites for immune priming, particularly for the induction of mucosal IgG and IgA antibody responses (50, 51). Indeed, intranasal immunization of MVA-Spike-dmLT-A elicited potent mucosal vaccine-specific IgA responses measured in BAL fluid. This is particularly encouraging, as secretory IgA plays a central role in protecting against respiratory viruses such as SARS-CoV-2 by neutralizing the virus at its portal of entry (52). Overall, the strong immunogenicity of MVA-Spike-dmLT-A - despite the presumably localized expression of the adjuvant - may be driven by the preferential tropism of MVA for DCs. MVA is known to efficiently infect DCs, thereby supporting robust antigen cross-presentation and T cell priming (53). Because the immune-stimulatory activity of dmLT-A critically relies on its action in DC (7, 16, 19, 54), its expression within MVA-infected DCs likely constitutes the key mechanism of immune activation. Co-encoding dmLT-A with MVA thereby ensures its targeted delivery to DC, maximizing efficacy through the dual effect of MVA’s DC tropism and the central role of DCs in mediating dmLT-A-driven responses. The intracellular cues driving DC activation and ultimately TH17 polarization are likely regulated by cAMP-PKA signaling classically induced by LT (22, 54). However, dmLT has also been shown to engage cAMP- and PKA-independent pathways, including inflammasome-associated signals, particularly in combination with TLR agonists such as MPL-A, thereby enhancing multifunctional CD4^+^ T cell responses (55). Which of these pathways are operative following MVA-dmLT-A immunization, and to what extent they shape the observed immune phenotype, remains to be determined.

A major finding of our study is that MVA-encoded dmLT-A dramatically improved safety outcomes compared to MVA-encoded dmLT. Intramuscular immunization with MVA-OVA-dmLT or MVA-Spike-dmLT caused noticeable local swelling (**Figures 1F**, **2C**), whereas MVA-Spike-dmLT-A did not induce swelling beyond that observed with MVA-Spike alone (**Figure 2C**). This was unexpected, because preclinical and clinical studies with dmLT adjuvant reported good safety and tolerability of the adjuvant through various routes (17, 29, 56). We have shown that only the full dmLT toxin but not the dmLT-A subunit alone was secreted when expressed by MVA (**Figure 2B**), suggesting that secreted dmLT may act on uninfected bystander cells and contribute to local inflammation. In contrast, MVA-expressed dmLT-A remains confined to infected cells, likely due to an endoplasmic reticulum retention signal normally masked in the AB_5_ toxin structure (57, 58). Successful secretion of the dmLT-A subunit may require the fully assembled dmLT complex. While tuning the expression of dmLT by employing different promoters could mitigate toxicity, this might compromise immunogenicity. MVA-dmLT-A circumvents this trade-off by maintaining strong immunogenicity without detectable toxicity.

This safety advantage was even more pronounced following intranasal administration. At higher doses, MVA-Spike-dmLT but not MVA-Spike-dmLT-A caused transient weight loss and a pronounced inflammatory response in the airways, correlating with elevated levels of pro-inflammatory cytokines (IL-1β, IL-6, IFN-γ, etc.; **Figure 5**) and chemokines in the lung shortly after immunization, which likely contributed to the transient illness (weight loss) observed (59). Notably, many of the early cytokines elevated by MVA-Spike-dmLT (IL-1β, IL-23, IL-17A, IL-22) are linked to TH17 responses (60); their high levels in the MVA-Spike-dmLT group at day 5 after immunization but muted levels in the MVA-Spike-dmLT-A group suggest that the presence of the B subunit accelerates and amplifies the initial innate immune activation. Despite this, both constructs induced comparable TH17 cell numbers by day 14, indicating that MVA-dmLT-A still provides the necessary cues for TH17 differentiation without triggering excessive cytokine release. These findings suggest that the initial priming event triggered by MVA encoding either dmLT or dmLT-A sets the trajectory for the subsequent antigen-specific immune response, including TH17 differentiation. However, the presence of secreted dmLT may gradually amplify unspecific inflammation through bystander activation, leading to broader cytokine release and transient pathology. In contrast, the expression of dmLT-A is restricted to the infected cell, which appears to limit this secondary wave of innate activation, resulting in a more focused and controlled immune environment. Importantly, the absence of any detectable adverse effects in the MVA-Spike-dmLT-A groups, even at high intranasal doses, indicates a substantial improvement in the therapeutic index of the vaccine. This finding aligns with other research showing that intranasal LT-A1 adjuvant did not cause the neurological side effects associated with holotoxin-based adjuvants (31).

Looking ahead, incorporating inbuilt adjuvants into viral vaccine vectors - and potentially into other platforms such as mRNA/LNP vaccines - could enable more precise tailoring of immune responses against mucosal pathogens. To our knowledge, this is the first report to integrate the dmLT-A gene into a viral vaccine vector and directly compare it to the holotoxin analog *in vivo*. Conventional MVA vectors expressing stabilized or multimeric forms of Spike have previously been employed, either in heterologous prime–boost regimens or in combination with conventional adjuvants (61–65). In contrast to these studies, our approach uniquely incorporates a genetically encoded adjuvant directly into the MVA vector. This design enhances both systemic and mucosal immune responses without the need for external adjuvants or complex delivery strategies, distinguishing our platform from existing MVA-based SARS-CoV-2 vaccine candidates. By combining the strong immunogenic profile of MVA with the safe mucosal-immune-modulating properties of dmLT-A, we present a highly attractive platform for vaccine development.

## Data Availability

The raw data supporting the conclusions of this article will be made available by the authors, without undue reservation.

## Supplementary material

Supplementary Figure 1 Vero cells were infected with 5 InfU per cell of MVA-Spike, MVA-Spike-dmLT, or MVA-Spike-dmLT-A for 1 hour. Cell lysates and cell culture supernatants were harvested 23 hours after infection and analyzed by Western blot for dmLT-A+B.

Supplementary Figure 2 **(A)** Fourteen days after prime immunization with 10^7^ InfU of the indicated recombinant MVAs, splenocytes were isolated and restimulated with MVA B8R peptide (TSYKFESV) on IFN-γ ELISPOT plates. IFN-γ-producing cells were revealed with biotinylated anti-IFN-γ antibody, followed by streptavidin-HRP and AEC substrate. To detect IL-17A-producing cells, splenocytes or lung cells were restimulated with Spike peptide pool on IL-17A ELISPOT plates; spots were developed with biotinylated anti-IL-17A antibody, followed by streptavidin-ALP and BCIP/NBT-plus substrate (Mabtech). Spots were counted using an ELISPOT reader. **(B)** Representative 20x tile scan images of immunohistochemically stained lung sections (day 12 post-immunization) show CD4 (red), B220 (green), and nuclei (blue). Scale bar = 1000 µm **(C)** Close-up views of representative lung sections highlight selected areas to facilitate visualization of iBALT. Scale bar = 250 µm.

Supplementary Figure 3 Wild-type C57BL/6J mice were immunized intranasally with 2,5×10^7^ InfU of MVA-Spike, MVA-Spike-dmLT, MVA-Spike-dmLT-A, or 5 µg dmLT protein + 5 µg Spike protein, or TBS (control). BAL was collected 5 days post-immunization and analyzed by a 48-plex Luminex assay. The heatmap shows log_10_-transformed concentrations (pg/mL) of all analytes measured, grouped by functional category (cytokines, chemokines, growth factors) according to the manufacturer.

Supplementary Figure 4 **(A)** Representative 20x tile scan images of immunohistochemically stained lung sections (day 5 post-immunization) of TBS immunized mice showing nuclei (blue) and specific staining of CD3 (red), and Ly6G (green). Scale bar = 1000 µm **(B)** Close-up view of a representative lung section highlighting a selected area with no detectable cell infiltration. Scale bar = 250 µm.
