# Supplementary figures and images for: MVA-Spike encoding the A subunit of dmLT safely enhances systemic and mucosal immune responses
Source: Front Immunol. 2026 Feb 5;17:1771410. doi: 10.3389/fimmu.2026.1771410 (PMC12916580; doi:10.3389/fimmu.2026.1771410)

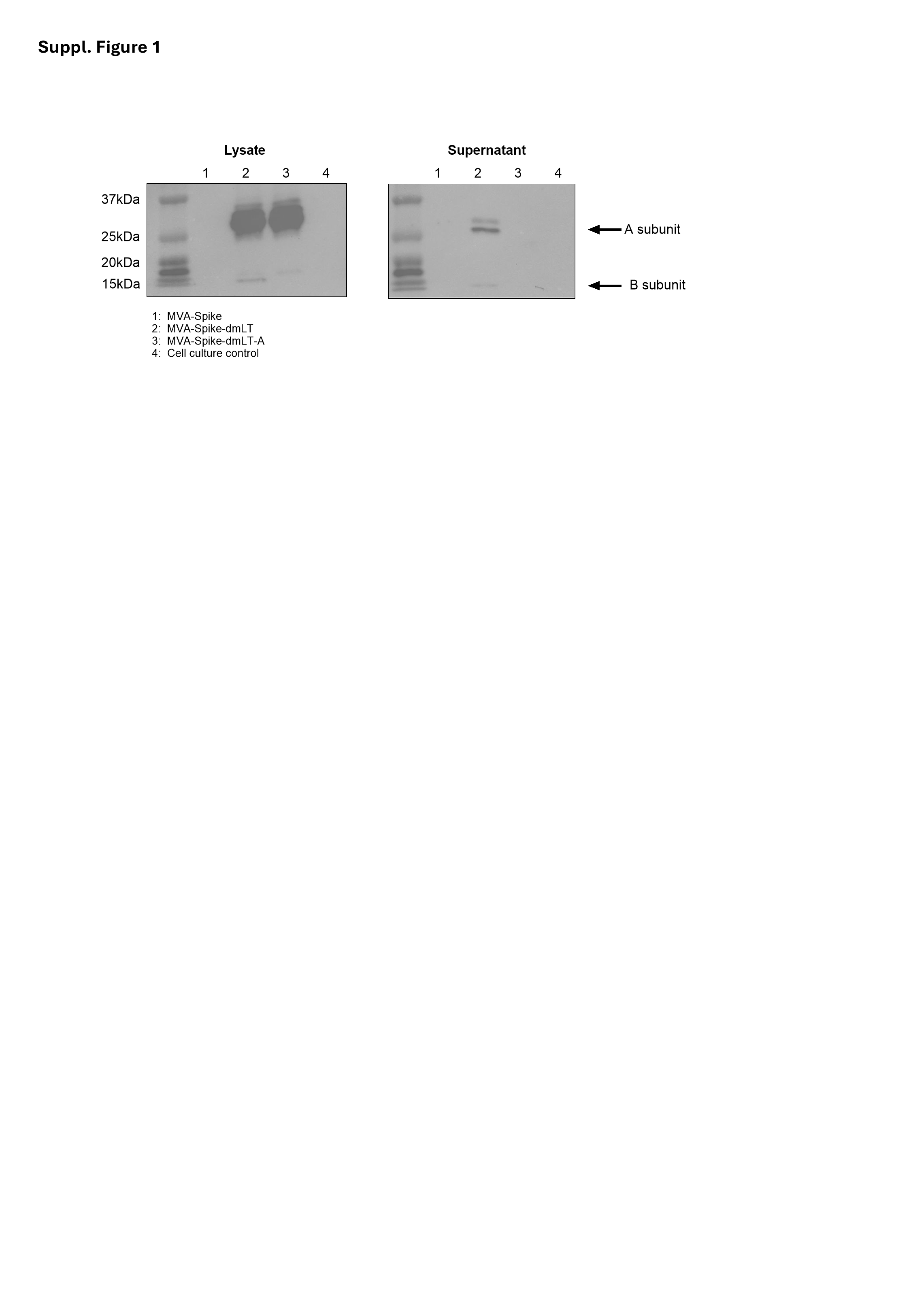

Supplement: Supplementary Figure 1 — Vero cells were infected with 5 InfU per cell of MVA-Spike, MVA-Spike-dmLT, or MVA-Spike-dmLT-A for 1 hour. Cell lysates and cell culture supernatants were harvested 23 hours after infection and analyzed by Western blot for dmLT-A+B. [file Image1.tif]

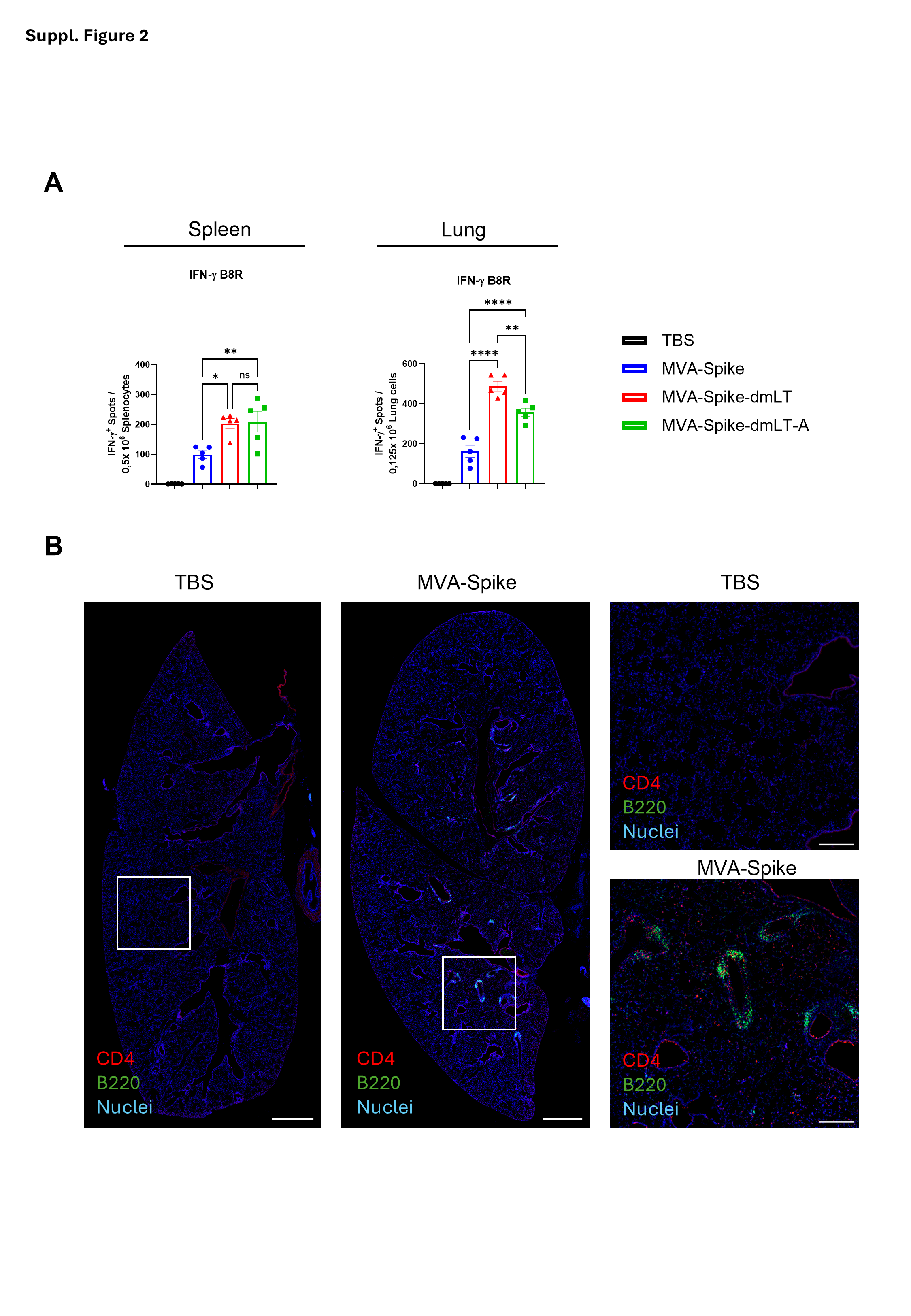

Supplement: Supplementary Figure 2 — (A) Fourteen days after prime immunization with 107 InfU of the indicated recombinant MVAs, splenocytes were isolated and restimulated with MVA B8R peptide (TSYKFESV) on IFN-γ ELISPOT plates. IFN-γ-producing cells were revealed with biotinylated anti-IFN-γ antibody, followed by streptavidin-HRP and AEC substrate. To detect IL-17A-producing cells, splenocytes or lung cells were restimulated with Spike peptide pool on IL-17A ELISPOT plates; spots were developed with biotinylated anti-IL-17A antibody, followed by streptavidin-ALP and BCIP/NBT-plus substrate (Mabtech). Spots were counted using an ELISPOT reader. (B) Representative 20x tile scan images of immunohistochemically stained lung sections (day 12 post-immunization) show CD4 (red), B220 (green), and nuclei (blue). Scale bar = 1000 µm (C) Close-up views of representative lung sections highlight selected areas to facilitate visualization of iBALT. Scale bar = 250 µm. [file Image2.tif]

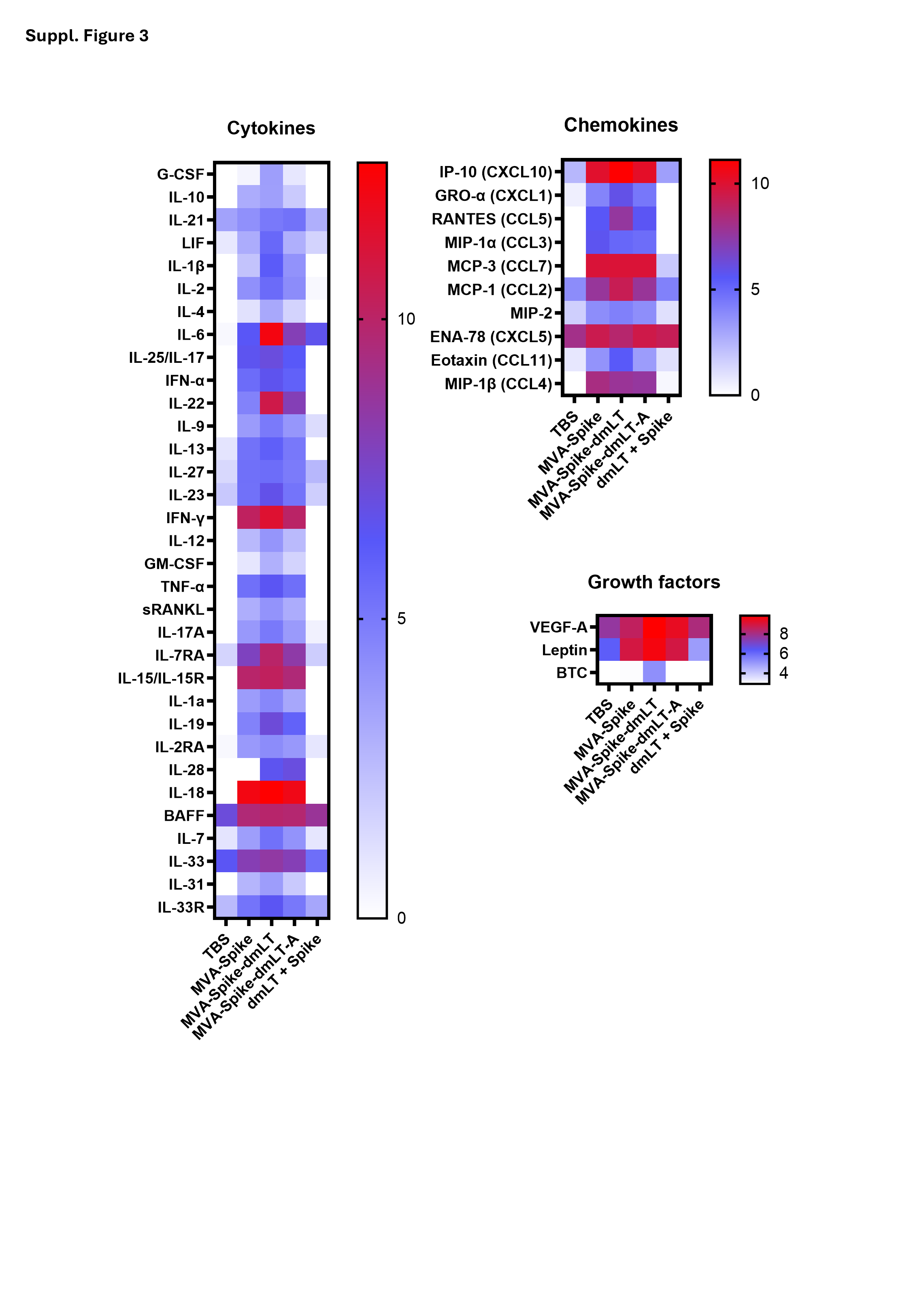

Supplement: Supplementary Figure 3 — Wild-type C57BL/6J mice were immunized intranasally with 2,5×10^7^ InfU of MVA-Spike, MVA-Spike-dmLT, MVA-Spike-dmLT-A, or 5 µg dmLT protein + 5 µg Spike protein, or TBS (control). BAL was collected 5 days post-immunization and analyzed by a 48-plex Luminex assay. The heatmap shows log10-transformed concentrations (pg/mL) of all analytes measured, grouped by functional category (cytokines, chemokines, growth factors) according to the manufacturer. [file Image3.tif]

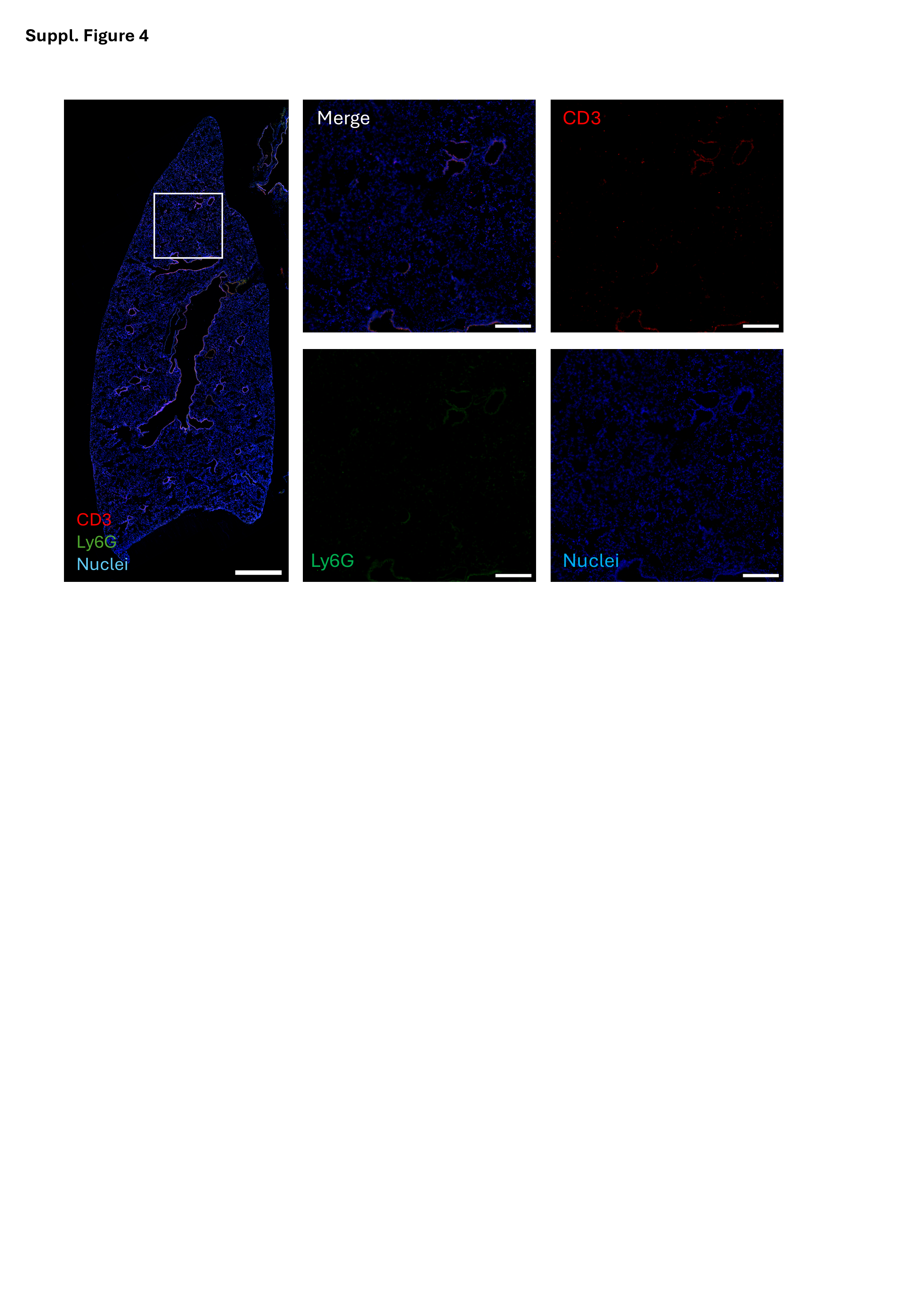

Supplement: Supplementary Figure 4 — (A) Representative 20x tile scan images of immunohistochemically stained lung sections (day 5 post-immunization) of TBS immunized mice showing nuclei (blue) and specific staining of CD3 (red), and Ly6G (green). Scale bar = 1000 µm (B) Close-up view of a representative lung section highlighting a selected area with no detectable cell infiltration. Scale bar = 250 µm. [file Image4.tif]
